# Effect of Lycopene Intake on the Fasting Blood Glucose Level: A Systematic Review with Meta-Analysis

**DOI:** 10.3390/nu15010122

**Published:** 2022-12-27

**Authors:** Takuro Inoue, Kazutaka Yoshida, Erika Sasaki, Koichi Aizawa, Hiroharu Kamioka

**Affiliations:** 1 Agricultural and Bio Resource Development Department, KAGOME Co., Ltd., 17 Nishitomiyama, Nasushiobara 329-2762, Japan; 2 Department of Ecological Symbiotic Science, Tokyo University of Agriculture, 1-1-1 Sakuragaoka, Setagaya-ku, Tokyo 156-8502, Japan; 3 Diet & Well-Being Research Department, KAGOME Co., Ltd., 17 Nishitomiyama, Nasushiobara 329-2762, Japan; 4 Health & Wellness Business Department, KAGOME Co., Ltd., Nihonbashi-Hamacho F-Tower, 3-21-1 Nihonbashi-Hamacho, Chuo-ku, Tokyo 103-8461, Japan

**Keywords:** lycopene, fasting blood glucose, diabetes mellitus, systematic review, meta-analysis

## Abstract

Lycopene is a lipophilic unsaturated carotenoid exhibiting a strong singlet oxygen-quenching ability. Herein, we investigated the effect of lycopene intake on the fasting blood glucose (FBG) level by conducting a systematic review and meta-analyses. We searched 15 databases (from the earliest date to June 2022 for PubMed or to August or September 2018 for the other databases) and included human interventional studies that assessed the effects of oral lycopene intake on FBG levels of participants ≥ 18 years of age. Three authors independently selected applicable studies and then assessed the study quality. Data were pooled as standardized mean difference (SMD) and analyzed by the random-effects model. Heterogeneity was assessed by I^2^ statistics. A meta-analysis including 11 trial arms (*n* = 750) revealed a tendency towards a significant decrease in FBG level with not-important heterogeneity [SMD = −0.15 (95% CI: −0.31, 0.00), *p* = 0.05, I^2^ = 9%]. Subgroup meta-analysis including two studies (*n* = 152) in type 2 diabetes patients revealed significantly decreased FBG levels with not-important heterogeneity [SMD = −0.37 (95% CI: −0.69, −0.05), *p* = 0.02, I^2^ = 0%]. Most studies meeting the eligibility criteria had a moderate risk of bias. The funnel plot for FBG suggested an absence of publication bias. In conclusion, this systematic review and meta-analyses suggested that lycopene intake exerted an FBG-decreasing effect.

## 1. Introduction

Type 2 diabetes (T2D) is a chronic metabolic disease characterized by high blood glucose levels, causing serious damage to the cardiovascular, renal, respiratory, as well as other systems [1]. The global diabetes prevalence is currently rising and has been estimated to be 10.9% (700 million people) by 2045 [2], while the global health expenditure for diabetes is expected to reach USD 776 billion in 2045 [3]. Therefore, preventing the initiation and progression of T2D is a critical global issue.

Glycemic control is one of the most important approaches to treating T2D [4], and the cornerstone of T2D treatment is a healthy lifestyle, which includes the adoption of a healthy diet, increased physical activity, maintenance of healthy body weight, and a smoking cessation plan [3]. Th glycemic index (GI) introduced in 1981 [5] and glycemic load (GL) based on GI [6] are well-known indices to estimate the postprandial blood glucose level rise, and some systematic reviews reported the usefulness of low GI diets and/or low GL diets for diabetes mellitus patients. Ojo et al. reported that low GI diets were more effective in controlling FBG and Hemoglobin A1c (HbA1c) than higher GI diets in T2D patients [7]. Chiavaroli et al. reported that low GI and/or GL diets reduced FBG and HbA1c in comparison with higher GI and/or GL diets in type 1 and type 2 diabetes patients [8]. Recently, the intake of antioxidant-rich foods is also recommended as part of the lifestyle [9] since oxidative stress is considered a major characteristic of the pathogenesis and development of T2D [10]. The total antioxidant capacity of the diet was suggested to play a role in reducing the risk of T2D in middle-aged women [11], and fasting blood glucose (FBG) levels were found to be significantly lower in T2D patients with a better oxidative balance score [12]. Some systematic reviews have also demonstrated that the intake of fruits and/or vegetables is inversely associated with the risk of T2D [13,14,15]. Since fruits and vegetables are rich in vitamins, flavonoids, and carotenoids, these antioxidants can be expected to play an important role in controlling the glycemic condition and/or providing a defense against T2D by reducing oxidative stress.

Lycopene is a lipophilic unsaturated carotenoid found in red-colored fruits and vegetables, including tomatoes, watermelon, red grapefruit, papaya, apricot, and guava. It exhibits a strong singlet oxygen-quenching ability, which is twice as high as that of beta-carotene and 100 times higher than that of alpha-tocopherol as a physical quenching rate [16]. Lycopene has been reported to exert beneficial effects in preventing many diseases, for example, cancer [17], cardiovascular diseases [18], diabetes mellitus [19], skin diseases [20], bone diseases [21], etc. Regarding antidiabetic effects, a higher dietary lycopene intake has been observed in non-T2D men compared to T2D men [22]. Increased plasma or serum lycopene levels have been reported to be associated with lower risks of T2D [23] and also better glycemic control (lower FBG) in T2D patients [24,25]. Recently, a review article summarized the lycopene effects on glycemic control in T2D. However, it was a narrative review, and a comprehensive literature search was not yet performed [19].

Several systematic reviews were conducted trying to evaluate the effect of tomato and/or its components on the FBG level. One systematic review with meta-analysis reported no significant difference in the FBG level between the tomato intervention and control groups [26]. Regarding lycopene, two systematic reviews did not address the effects on the FBG level because of inconsistency [27] or data unavailability of the included studies [28]. In these systematic reviews, possible limitations include the fact that only two to four electronic bibliographic databases were used for the literature search [26,27,28], and eligible studies were restricted to English or other Germanic/Romanic languages [27,28]. Therefore, there is a need for a more exhaustive literature search to find studies listed in other databases and/or reported in languages other than English and Germanic/Romanic. In this study, we performed a systematic review with meta-analysis to summarize the evidence relative to the effect of lycopene intake on the FBG level that was collected in human interventional trials, using more bibliographic databases and without restricting the study eligibility criteria by language.

## 2. Materials and Methods

### 2.1. Protocol and Registration

This systematic review and meta-analyses were conducted with the research question “Does oral lycopene intake improve FBG level, one of the most important biomarkers of diabetes mellitus, in participants ≥ 18 years of age?” and reported in accordance with the PRISMA 2009 statement [29]. The protocol was registered with PROSPERO, the International Prospective Register of Systematic Reviews, before starting the review (Registration number CRD42018104595).

### 2.2. Literature Search

We searched the following 15 databases: PubMed (MEDLINE), Web of Science (Core Collection, FSTA, Derwent Innovations Index, Medline, Zoological Record, BIOSIS Citation Index, Current Chemical Reactions, Data Citation Index, Current Contents Connect, Index Chemicus), Cochrane Library, SciFinder, Global Index Medicus, Western Pacific Region Index Medicus, CINAHL, Reaxys, Ichushi-Web, JDream III (JMEDPlus), AGRIS, University Hospital Medical Information Network-Clinical Trials Registry, International Clinical Trials Registry Platform, ClinicalTrials.gov, and PROSPERO. We searched PubMed (MEDLINE) from the earliest date to June 2022 and the other databases from each earliest date to August or September 2018. The database search strategy is presented in Supplementary Table S1. We also searched the reference lists of the included relevant papers and the latest reviews.

### 2.3. Study Selection

Studies were selected similar to a previous report [30]. Based on the research question, we set the selection criteria as follows: (A) participants were ≥18 years of age; (B) intervention was the oral intake of test foods containing lycopene; (C) control was the oral intake of test foods not containing lycopene, oral intake of test foods containing lower levels of lycopene than intervention, or nothing; (D) outcome was FBG level; and (E) study design was a randomized controlled parallel trial (RCT-P), quasi-RCT-P, non-RCT-P, randomized controlled crossover trial (RCT-C), quasi-RCT-C, or non-RCT-C. For the studies retrieved from the literature search, three authors (K.Y., E.S., and K.A.) independently reviewed the titles and abstracts of them to identify studies that potentially met the selection criteria and reviewed the full text of selected studies to assess their eligibility. If there was any uncertainty or disagreement about eligibility, it was discussed with another author (T.I.) and resolved. Proceedings, grey literature, and unpublished studies were excluded. Eligibility was not restricted by language.

### 2.4. Data Extraction

We extracted data from the included studies similar to a previous report [30] for quality assessment and evidence synthesis, using a standardized, pre-piloted form. Extracted data included: citation, author, title, objective, setting, trial registration identifier, participant characteristics, intervention conditions, control conditions, outcomes, study design, randomization, blinding (participant, care provider, and outcome assessor), number of randomized participants, number of analyzed participants, results, conclusion, adverse events, cost of intervention, and funding. We extracted the mean and standard deviation (SD) values for FBG before and after the intervention. We also extracted the mean difference and SD between values before and after the intervention. When the SD values of the mean difference were not reported, we calculated them using the formula: square root [(SD_before_)^2^ + (SD_after_)^2^ − 2R × SD_before_ × SD_after_], assuming a correlation coefficient R = 0.5 [31]. The unit of FBG level was represented in mg/dL; if the values were originally published in mmol/L, they were converted to mg/dL by multiplying a factor of 18. Three authors (K.Y., E.S., and K.A.) independently extracted data, and any discrepancies were discussed with another author (T.I.) and resolved. If necessary, missing data were requested from the study authors via e-mail.

### 2.5. Quality Assessment

Three authors (K.Y., E.S., and K.A.) independently assessed the risk of bias in the reviewed studies, similar to a previous report [30], using a modified checklist of the Cochrane Handbook [32]. Briefly, the checklist included 13 items as follows: (A) randomization; (B) concealment of allocation; (C) blinding of participants; (D) blinding of care providers; (E) blinding of outcome assessors; (F) rate of drop-out; (G) intention-to-treat analysis; (H) selective outcome reporting; (I) similarity of baseline; (J) co-intervention; (K) compliance; (L) outcome assessment timing; and (M) other potential bias source. We scored each item as “there is no risk of bias” (+), “there is a risk of bias”, or “unclear” (−). Based on the total number of (−), we evaluated each study as follows: 0–3, low risk of bias; 4–8, moderate risk of bias; 9–13, high risk of bias. If there were any uncertainties and disagreements on the risk of bias, they were discussed with another author (T.I.) and resolved.

### 2.6. Statistical Analysis

We conducted meta-analyses similar to a previous report [30] using Review Manager (RevMan Version 5.3 for Windows, The Cochrane Collaboration, Copenhagen, Denmark). We used the mean difference and its SD values to evaluate the intervention effect. To include studies with more than two intervention groups in meta-analyses, we combined relevant intervention groups using a standard formula [31] to create single pair-wise comparisons. To compare effect sizes across studies, we used the standardized mean differences (SMDs) with 95% CI as a summary statistic. The random-effects model [33] was used to calculate the pooled SMDs, and a two-sided *p*-value < 0.05 was considered statistically significant. Heterogeneity was evaluated in the Forest plot [34] according to the I^2^ statistics defined as follows: 0–40%, not-important; 30–60%, moderate; 50–90%, substantial; and 75–100%, considerable [31]. We also evaluated the inconsistency of evidence according to the I^2^ statistics. We evaluated the publication bias by visual inspection of a funnel plot.

### 2.7. Subgroup Analysis

To investigate the factors that influenced the effect of lycopene on FBG and potential sources of heterogeneity, we planned in advance to conduct the subgroup analyses on the following viewpoints: (A) study design (focused on RCT-P); (B) types of test foods (supplement type or others); (C) length of the intervention period (shorter period or longer period); (D) lycopene level in test foods (lower level or higher level); and (E) participants’ characteristics (healthy or others). Additionally, the following subgroup analysis was conducted post hoc: separating studies by participants’ characteristics (diabetes mellitus participants and others).

## 3. Results

### 3.1. Search Results

**Figure 1:**
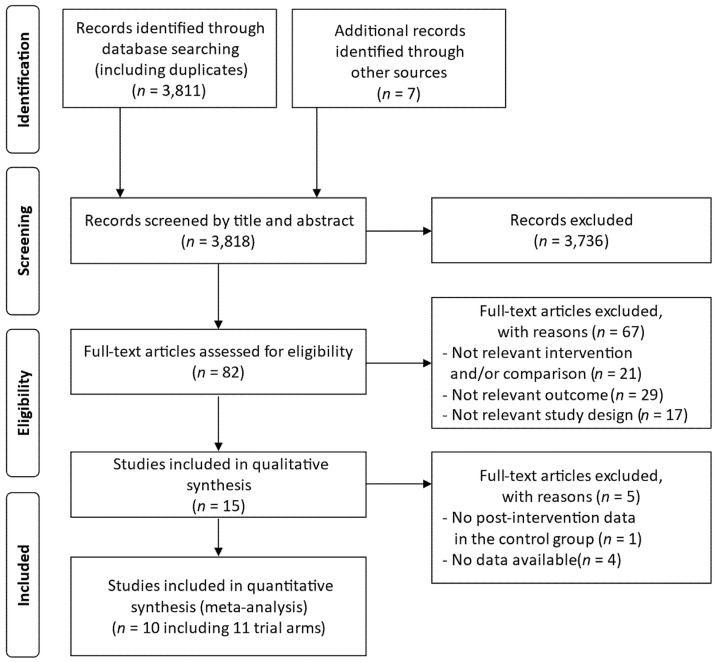
Flow diagram of the study selection process.

The results of the study selection process are described in Figure 1. The literature searches (database search and additional sources search) yielded 3818 records, including duplicates, of which 15 studies met the eligibility criteria and were qualitatively assessed for risk of bias (Table 1) [35,36,37,38,39,40,41,42,43,44,45,46,47,48,49]. Ten of these 15 studies were included in the meta-analysis (Table 2) [36,37,39,41,42,44,45,46,47,49], and five studies were excluded due to either no post-intervention data in the control group or no data available (Table 3) [35,38,40,43,48]. Of the 15 studies, 13 were reported in English, and the others were reported either in Chinese (*n* = 1) [42] or Russian (*n* = 1) [36].

### 3.2. Study Characteristics

The study characteristics for the 15 studies that met the eligibility criteria are described in Table 2 and Table 3. Study locations included Russia (*n* = 3), Greece (*n* = 2), Japan (*n* = 2), Israel (*n* = 1), USA (*n* = 1), UK (*n* = 1), China (*n* = 1), France (*n* = 1), New Zealand (*n* = 1), Iran (*n* = 1), and Korea (*n* = 1). Ten studies used an RCT-P [35,36,39,40,41,42,45,47,48,49], one used an RCT-C [46], three used a non-RCT-P [38,43,44], and one used a non-RCT-C design [37]. In six studies, participants were healthy subjects [39,40,45,46,47] and ultra-marathon runners [43]. In the other studies, participants were moderately overweight [41], obese [48,49], or had a metabolic syndrome [44], grade-1 hypertension [37], or type 2 diabetes [35,36,38,42]. For test foods, nine studies used either tomato extract capsules or lycopene supplements or synthetic lycopene capsules [36,37,38,39,40,41,42,45,48], three studies used tomato juice [35,43,44], one study used semi-dried tomatoes [47], one study used lycopene-enriched ice cream [46], one study used lycopene-enriched dark chocolate [48], one study used a tomato-rich diet [41], and one study used carrot and kale juice and carrot and cabbage juice [49]. The dosages of lycopene ranged from 6 to 50 mg/day, and intake periods ranged from 2 weeks to 6 months.

### 3.3. Quality Assessment of the Studies

Of the 15 studies considered, one study [47] was assessed as having a low risk of bias, while 13 studies [35,36,37,38,39,40,41,42,44,45,46,48,49] were assessed as having a moderate risk of bias, and one study [43] was assessed as having a high risk of bias (Table 1). In most studies, randomization, concealment of allocation, blinding of care provider, intention-to-treat analysis, selective outcome reporting, compliance, and outcome assessment timing were not reported in detail (Table 1).

### 3.4. Meta-Analysis

**Figure 2:**
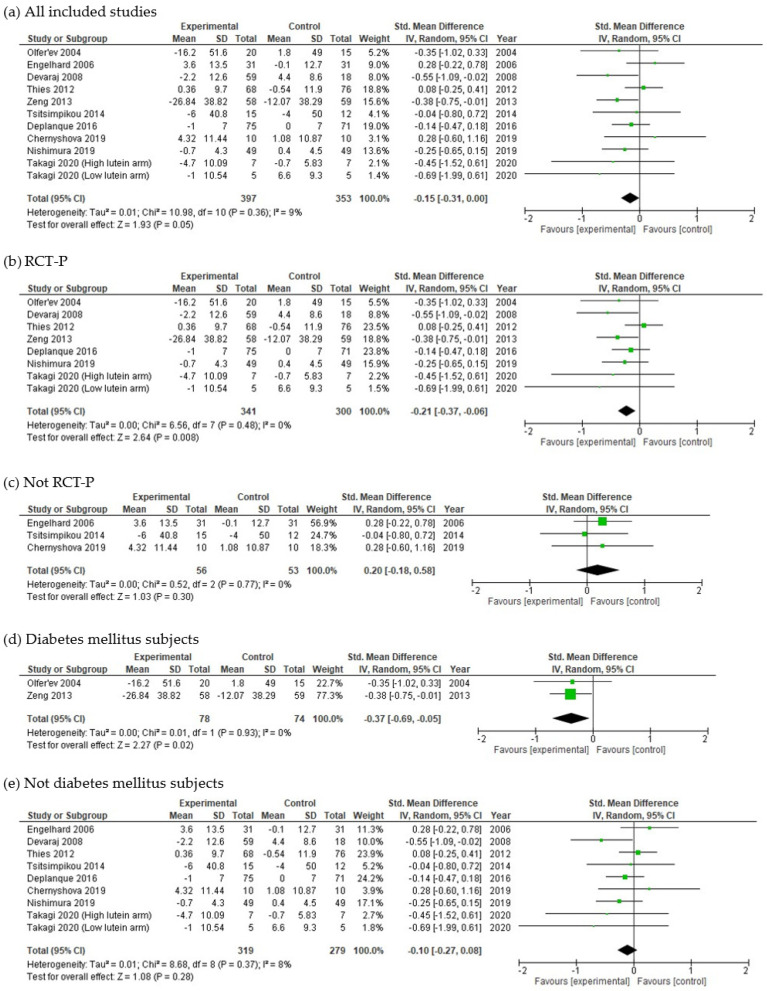
Meta-analysis and subgroup meta-analyses of the effects of lycopene on fasting blood glucose (FBG): (**a**) all included studies (*n* = 11 trial arms), (**b**) RCT-P (*n* = 8 trial arms), (**c**) not RCT-P (*n* = 3 studies), (**d**) diabetes mellitus subjects (*n* = 2 studies), and (**e**) not diabetes mellitus subjects (*n* = 9 trial arms). The green squares represent the standardized mean difference in each study. The black diamonds represent the pooled effects in each meta-analysis. RCT-P, randomized controlled parallel trial; Std., standardized; SD, standard deviation; IV, inverse variance; CI, confidence interval [36,37,39,41,42,44,45,46,47,49].

The meta-analysis, which included 10 studies (11 trial arms) with a total of 750 participants, revealed a tendency towards a significant decrease in FBG in the lycopene group compared with the control group [SMD = −0.15 (95% CI: −0.31, 0.00), *p* = 0.05], and heterogeneity was not important (I^2^ = 9%) (Figure 2a).

Some of the subgroup meta-analyses revealed a significant decrease in FBG in the lycopene group compared to the control group. The RCT-P study design, which included 7 studies (8 trial arms) with a total of 641 participants, revealed a significantly decreased FBG with not-important heterogeneity [SMD = −0.21 (95% CI: −0.37, −0.06), *p* = 0.008, I^2^ = 0%] (Figure 2b). The other study designs, which included 3 studies with a total of 109 participants, revealed no significant change in FBG with not-important heterogeneity [SMD = 0.20 (95% CI: −0.18, 0.58), *p* = 0.30, I^2^ = 0%] (Figure 2c). The T2D participants, which included 2 studies with a total of 152 T2D participants, exhibited a significantly decreased FBG with not-important heterogeneity [SMD = −0.37 (95% CI: −0.69, −0.05), *p* = 0.02, I^2^ = 0%] (Figure 2d). Participants other than T2D, which included 8 studies (9 trial arms) with a total of 598 participants, showed no significant change in FBG with not-important heterogeneity [SMD = −0.10 (95% CI: −0.27, 0.08), *p* = 0.28, I^2^ = 8%] (Figure 2e). The other subgroup meta-analyses revealed no significant changes in FBG in the lycopene group compared with the control group (Supplementary Figure S1). Subgroup meta-analysis for lycopene levels in test foods could not be conducted due to a lack of lycopene dose information.

### 3.5. Publication Bias

**Figure 3:**
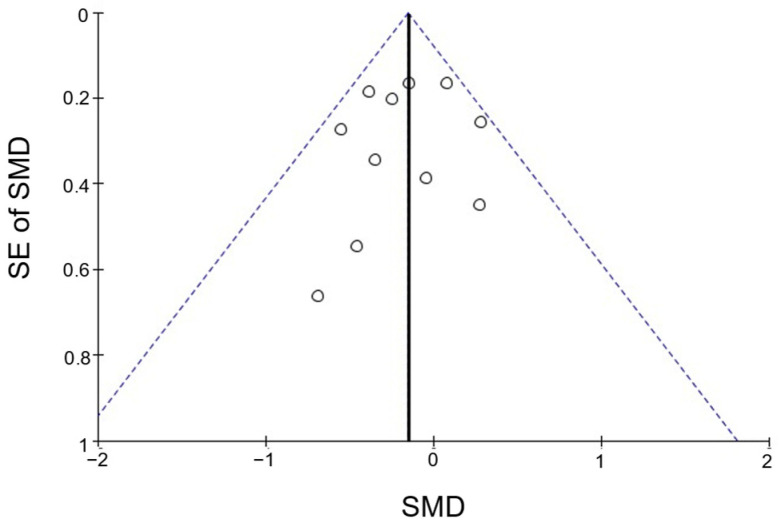
Funnel plot of studies included in the meta-analysis on fasting blood glucose (*n* = 11 trial arms). The vertical solid line represents the pooled effect size, and the dashed lines represent the 95% confidence interval. SMD, standardized mean difference; SE, standard error.

The funnel plot for FBG suggested an absence of publication bias (Figure 3). In subgroup analyses, assessments of publication bias were not meaningful because too few studies were included.

## 4. Discussion

### 4.1. Effects of Lycopene on FBG

The meta-analysis with all included studies revealed a tendency towards a significant decrease in the FBG level, and the subgroup meta-analysis restricted to T2D patients suggested a significant decrease in the FBG level in the lycopene group compared with that in the control group. To the best of our knowledge, this is the first systematic review supporting the indication that lycopene improves the FBG level in T2D patients.

Our literature search and study selection using 15 bibliographic databases without restricting the study eligibility criteria by language was extended to studies in Chinese and Russian, with qualities that were similar to those of studies in English. A Chinese study in T2D patients reported that lycopene intake (30 mg/day for 6 months) significantly improved the FBG level compared to the pre-intake level [42]. A Russian study in T2D patients disclosed no significant effect of lycopene intake (30 mg/day for 12 weeks) on the FBG level, although the decrease of the FBG level in the lycopene group was larger than that in the control group [36]. Both studies were not included in the previous systematic reviews [26,27,28]. Therefore, this systematic review could provide novel insights by including those studies. In this study, we excluded one study [50] included in the previous systematic review [26] because the control intervention did not meet our study selection criteria (comparison between polyphenol-enriched tomato juice and standard tomato juice). Although the difference in the included studies among these systematic reviews might be due to the differences in the literature search strategy and detailed study eligibility criteria, the methodological quality of each systematic review should be assessed using critical appraisal instruments, such as AMSTAR2 [51].

In this systematic review, several pre-set subgroup meta-analyses and one post hoc subgroup meta-analysis were conducted, and two subgroup meta-analyses restricted to RCT-P and T2D patients revealed a significantly decreased FBG with not-important heterogeneities. A randomized controlled trial is generally the highest quality study design and the gold standard in interventional clinical trials [52], while non-randomized controlled trials might have a potentially higher risk of bias. In this study, the subgroup meta-analysis restricted to RCT-P (excluding not-RCT-P) disclosed a significantly decreased FBG. Although the meta-analysis with all included studies revealed only a tendency towards a significant decrease in the FBG level, a significant decrease might be confirmed by reporting more high-quality primary studies.

For studies included in the meta-analyses, the average FBG level in the pre-intake period in T2D patients was much higher than that in not-T2D patients (161.7 mg/dL vs. 93.4 mg/dL), which was assumed to be the reason why the effect of lycopene was exerted only in T2D patients. In food studies, it is often difficult to identify appropriate placebo foods when using complex test foods. However, the studies in T2D patients included in the meta-analyses used supplement-type test foods such as tomato extract capsules [36] and lycopene capsules [42]. Therefore, the effects of ingredients other than lycopene can be expected to be small, although they could not be completely ruled out. The effects of medications should be considered when patients participate in interventional clinical studies. In T2D patients studies included in the meta-analyses [36,42], the T2D participants had received medications. However, the effects of medications seemed to be small because the drugs prescribed prior to the study were continued throughout the study in both the intervention and the control group [36], or there was no significant difference in medication status between the two groups [42].

Therefore, lycopene intake can be expected to have a positive effect on FBG levels even under medication, although limited to individuals with high FBG levels. However, two studies [35,38] with T2D participants were excluded from the meta-analyses due to the data unavailability for this study, and additional primary studies are needed to clarify the FBG-improving effect of lycopene.

### 4.2. Effect Size and Possible Mechanisms

In both two studies with T2D patients, the dosage of lycopene was 30 mg/day, and a meta-analysis restricted to these studies showed that the SMD for FBG was −0.37 (95% CI: −0.69, −0.05), that is, the mean difference for FBG was −15.25 (95% CI: −28.15, −2.35) mg/dL, which was about 9.4% of the FBG level at the pre-intake point. Gao et al. conducted a cross-sectional study to determine whether increased carotenoids intake was associated with a reduced risk of gestational diabetes mellitus and reported an inverse association between lycopene intake and FBG; each 1 mg increase in lycopene intake was associated with a 0.09 mg/dL decrease in FBG [53]. However, it is difficult to simply compare these results due to differences in participants and study designs. Some foods other than tomatoes have been reported to have FBG-improving effects in T2D patients. Shabani et al. reported that garlic intake improved FBG levels, and its effect size was −10.90 mg/dL (95% CI: −16.40, −5.40) in a systematic review [54]. Suksomboon et al. reported that *Aloe vera* intake also improved FBG levels, and its effect size was −21.06 mg/dL (95% CI: −42.3, 0.00) in a systematic review [55]. Therefore, lycopene intake can be expected to have an effect similar to those of garlic and *Aloe vera* in improving FBG levels and to provide better FBG control in combination with these foods. In addition, if the FBG-improving effect size of each food and their combination could be clearly shown, it would be an easy-to-understand guideline for T2D patients to try them in their diet and further expected to be reflected in the standards of diet treatment in diabetes.

Previous studies have suggested some possible mechanisms by which lycopene affects FBG levels. Hashimoto et al. examined the effect of lycopene on glucose tolerance in normal rats and found that a lycopene-rich tomato intake improved glucose tolerance via an increase in plasma leptin levels that enhanced insulin sensitivity [56]. Some reports using diabetic model rats indicated the importance of the antioxidative effect of lycopene. Yin et al. reported that lycopene intervention decreased the FBG level in T2D model rats, and lycopene might improve glucose metabolism by reducing oxidized low-density lipoprotein cholesterol [57]. Zheng et al. described that lycopene intervention decreased the FBG level in a dose-dependent manner in T2D rats and concluded that lycopene protected against diabetic progression and prevented further complications of diabetic rats through ameliorating oxidative stress and inflammation as well as improving the systemic antioxidative capacity [58]. It is possible that lycopene affects FBG levels through multiple pathways, and further evidence, especially in human studies, needs to be accumulated.

### 4.3. Strengths and Limitations

The strengths of this study include an extensive literature search that used many databases not restricted by language. Although the latest additional literature search was conducted only in PubMed (MEDLINE), a basic search in 15 databases and additional search enabled us to find studies reported in languages other than English.

This study is not without limitations. Most included studies had a moderate risk of bias. Since any missing data could not be obtained from the study authors, we excluded studies with no data available and did not consider them in the meta-analyses. Although we imputed partly missing data according to the Cochrane Handbook [31], this procedure might have created a risk of bias. Since the protocol for this study was set in August 2018, this systematic review corresponded only partially to the latest PRISMA 2020 [59] and PRISMA-S [60] guidelines. In addition, the definite criteria to assess the strength of evidence were not set in advance or assessed in this study. The strength of evidence should be assessed using an appropriate instrument, for example, the GRADE approach [61]. In this study, we focused only on the FBG levels. However, other blood biomarkers (for example, insulin, homeostasis model of risk assessment-insulin resistance (HOMA-IR), Hemoglobin A1c and C peptide, etc.) should be evaluated to better understand the effects of lycopene on glucose metabolism.

## 5. Conclusions

This systematic review and meta-analysis adopted a more exhaustive literature search than the previous systematic reviews, that is, using 15 databases without restricting the study eligibility criteria by language, and highlighted an FBG-decreasing effect of lycopene intake, especially in T2D patients. In order to clarify this effect, additional clinical trials in T2D patients are needed, not only to evaluate the effect of lycopene on FBG but also on other glucose metabolism markers.

## Figures and Tables

**Table 1:** Quality assessment of the selected studies.

Selected Studies	Sources of Risk of Bias *													Total Number of “−”
	(A)	(B)	(C)	(D)	(E)	(F)	(G)	(H)	(I)	(J)	(K)	(L)	(M)	
Upritchard 2000 [35]	+	+	−	−	+	−	−	−	−	+	−	+	+	7
Olfer’ev 2004 [36]	−	−	+	−	+	−	−	−	+	+	−	−	+	8
Engelhard 2006 [37]	−	−	+	−	+	+	−	−	+	+	−	−	+	7
Neyestani 2007 [38]	−	−	+	+	−	+	+	−	+	+	−	−	+	6
Devaraj 2008 [39]	−	−	+	−	+	−	−	−	+	+	−	−	+	8
Kim 2011 [40]	−	−	+	−	+	−	−	−	+	+	−	−	+	8
Thies 2012 [41]	−	−	−	−	+	−	−	+	+	+	−	−	+	8
Zeng 2013 [42]	−	−	−	−	+	−	−	−	+	+	−	+	+	8
Samaras 2014 [43]	−	−	−	−	+	+	+	−	−	+	−	−	−	9
Tsitsimpikou 2014 [44]	−	−	−	−	+	+	+	−	+	+	−	−	+	7
Deplanque 2016 [45]	−	+	+	+	+	+	−	−	+	+	−	+	−	5
Chernyshova 2019 [46]	−	−	−	−	+	+	+	−	−	+	−	−	+	8
Nishimura 2019 [47]	+	+	+	+	+	−	−	+	+	+	+	+	+	2
Wiese 2019 [48]	−	−	+	−	+	+	+	−	−	+	−	−	+	7
Takagi 2020 [49]	−	−	+	−	+	−	−	−	−	+	−	+	+	8

+, “there is no risk of bias”; −, “there is a risk of bias” or “unclear”. * Sources of risk of bias corresponded to the following criteria: (A) randomization; (B) concealment of allocation; (C) blinding of participants; (D) blinding of care providers; (E) blinding of outcome assessors; (F) rate of drop-out; (G) intention-to-treat analysis; (H) selective outcome reporting; (I) similarity of baseline; (J) co-intervention; (K) compliance; (L) outcome assessment timing, and (M) other potential bias source. A larger number for “−” indicates a higher risk of bias.

**Table 2:** Characteristics of the included studies.

Sample Size, Sex	Participant, Age (Years)	Sample Size, Sex	Intervention/Control	Lycopene Dosage Per Day	Duration (Intake Period)	Outcome (Blood Biomarkers of Glucose Metabolism)	Study Design
Olfer’ev 2004 [36], Russia	Type 2 diabetic postmenopausal women, mean age 66.4	I: 20 (all F) C: 15 (all F)	I: Tomato extract capsule (3/day) C: Placebo capsule (3/day)	I: 30 mg C: 0 mg	12 weeks	FBG	RCT-P
Engelhard 2006 [37], Israel	Grade-1 hypertensive subjects, age range 30–73	I: 31 (13 F/18 M) C: 31 (13 F/18 M)	I: Tomato extract capsule (1/day) C: Placebo capsule (1/day)	I: 15 mg C: 0 mg	8 weeks	FBG	non-RCT-C
Devaraj 2008 [39], USA	Healthy subjects, age range ≥40	I1: 21 (17 F/4 M) I2: 17 (13 F/4 M) I3: 21 (14 F/7 M) C: 18 (14 F/4 M)	I1: Lycopene capsule (1/day) I2: Lycopene capsule (1/day) I3: Lycopene capsule (1/day) C: Placebo capsule (1/day)	I1: 6.5 mg I2: 15 mg I3: 30 mg C: 0 mg	8 weeks	FBG	RCT-P
Thies 2012 [41], UK	Moderate overweight subjects, age range 40–65	I1: 68 (40 F/28 M) I2: 81 (46 F/35 M) C: 76 (46 F/30 M)	I1: Low-tomato diet and tomato extract capsule (1/day) I2: High-tomato diet C: Low-tomato diet	I1: 10 mg I2: 32–50 mg C: 0.3 mg	12 weeks	FBG Insulin HOMA-IR QUICKI	RCT-P
Zeng 2013 [42], China	Type 2 diabetic patients, age range ≥60	I: 58 C: 59	I: Lycopene capsule (4/day) C: Placebo capsule (4/day)	I: 30 mg C: 0 mg	6 months	FBG PBG HbA1c	RCT-P
Tsitsimpikou 2014 [44], Greece	Metabolic syndrome subjects, mean age 54.9	I: 15 (2 F/13 M) C: 12 (1 F/11 M)	I: Tomato juice C: None	I: NA C: 0 mg	2 months	FBG Insulin FIRI	non-RCT-P
Deplanque 2016 [45], France	Healthy subjects, mean age 34.9	I: 75 C: 70	I: Tomato extract capsule (1/day) C: Placebo capsule (1/day)	I: 15 mg C: 0 mg	2 weeks	FBG	RCT-P
Chernyshova 2019 [46], Russia	Healthy subjects, mean age 33.4	I: 10 (5 F/5 M) C: 10 (5 F/5 M)	I: Lycopene-enriched ice cream (50 g/day) C: Ice cream (50 g/day)	I: 7 mg C: 0 mg	4 weeks	FBG	RCT-C
Nishimura 2019 [47], Japan	Healthy subjects, age range 30–70	I: 49 C: 49	I: Semidried high-lycopene tomato (50 g/day) C: Semidried lycopene-free tomato (50 g/day)	I: 22.0–27.8 mg C: 0 mg	12 weeks	FBG HbA1c HOMA-IR	RCT-P
Takagi 2020 [49], Japan	Obese men, age range 40–65	I1: 7 (all M) I2: 5 (all M) C1: 7 (all M) C2: 5 (all M)	I1: Carrot and kale juice (high lycopene + high lutein) (200 mL/day) I2: Carrot and cabbage juice (high lycopene + low lutein) (200 mL/day) C1: Carrot and kale juice (low lycopene + high lutein) (200 mL/day) C2: Carrot and cabbage juice (low lycopene + low lutein) (200 mL/day)	I1: 7.56 mg I2: 8.6 mg C1: 0 mg C2: 0 mg	8 weeks	FBG	RCT-P

I, intervention group; C, control group; F, female; M, male; FBG, fasting blood glucose; HOMA-IR, homeostasis model assessment-insulin resistance; QUICKI, Quantitative Insulin-Sensitivity Check Index; PBG, postprandial blood glucose; HbA1c, Hemoglobin A1c; FIRI, Fasting Insulin Resistance Index; RCT-P, randomized controlled parallel trial; RCT-C, randomized controlled crossover trial; NA, not available.

**Table 3:** Characteristics of the excluded studies.

Sample Size, Sex	Participant, Age (Years)	Sample Size, Sex	Intervention/Control	Lycopene Dosage Per Day	Duration (Intake Period)	Outcome (Blood Biomarkers of Glucose Metabolism)	Study Design	Reason for Exclusion
Upritchard 2000 [35], New Zealand	Type 2 diabetic patients, mean age 59	I1: 15 (5 F/10 M) I2: 12 (6 F/6 M) I3: 12 (6 F/6 M) C: 13 (3 F/10 M)	I1: Tomato juice (500 mL/day) I2: Vitamin E (800 U/day) I3: Vitamin C (500 mg/day) C: Placebo capsule (1/day)	I1: NA I2: 0 mg I3: 0 mg C: 0 mg	4 weeks	FBG HbA1c	RCT-P	No data available
Neyestani 2007 [38], Iran	Type 2 diabetic patients, mean age 54	I: 16 (9 F/7 M) C: 19 (10 F/9 M)	I: Lycopene supplement C: Placebo supplement	I: 10 mg C: 0 mg	8 weeks	FBG HbA1c	non-RCT-P	No data available
Kim 2011 [40], Korea	Healthy subjects, mean age 34.3	I1: 41 (all M) I2: 37 (all M) C: 38 (all M)	I1: Tomato extract capsule (1/day) I2: Tomato extract capsule (1/day) C: Placebo capsule (1/day)	I1: 6 mg I2: 15 mg C: 0 mg	8 weeks	FBG	RCT-P	No data available
Samaras 2014 [43], Greece	Ultra-marathon runners, mean age 44.9	I1: 15 (2 F/13 M) I2: 16 (2 F/14 M) C: 12 (all M)	I1: Tomato juice I2: Protein bar C: Carbohydrate supplementation beverage	I1: NA I2: NA C: NA	2 months	FBG	non-RCT-P	No post-intervention data in the control group
Wiese 2019 [48], Russia	Moderate obese subjects, mean age 55	I1: 6 (3 F/3 M) I2: 6 (3 F/3 M) C1: 6 (3 F/3 M) C2: 6 (3 F/3 M)	I1: Lycopene-enriched dark chocolate (10 g/day) I2: Lycopene capsule (1/day) C1: Dark chocolate (10 g/day) C2: Lycopene capsule (1/day)	I1: 7 mg I2: 30 mg C1: 0 mg C2: 7 mg	1 month	FBG	RCT-P	No data available

For abbreviations, see Table 2.

## Data Availability

The protocol of this study was registered with PROSPERO (Registration number CRD42018104595). More detailed information and the datasets generated and/or analyzed in this systematic review are available from the corresponding author upon reasonable request.

## Supplementary Materials

The following supporting information can be downloaded at: https://www.mdpi.com/article/10.3390/nu15010122/s1, Table S1: Search strategy for electronic bibliographic databases; Figure S1: Subgroup meta-analyses of the effects of lycopene on fasting blood glucose.
