# Supplementary material for: Effect of Lycopene Intake on the Fasting Blood Glucose Level: A Systematic Review with Meta-Analysis
Source: Nutrients. 2022 Dec 27;15(1):122. doi: 10.3390/nu15010122 (PMC9823324; doi:10.3390/nu15010122)
Supplement: Supplementary file 1 [file nutrients-15-00122-s001.zip › nutrients-2083337_Supplementary Figure S1.pdf]

(a) Supplement -type test foods

Study or Subgroup	Experimental Mean	Experimental SD	Experimental Total	Control Mean	Control SD	Control Total	Weight	Std. Mean Difference IV, Random, 95% CI	Year
Olfer'ev 2004 [36]	-16.2	51.6	20	1.8	49	15	9.0%	-0.35 [-1.02, 0.33]	2004
Engelhard 2006 [37]	3.6	13.5	31	-0.1	12.7	31	13.9%	0.28 [-0.22, 0.78]	2006
Devaraj 2008 [39]	-2.2	12.6	59	4.4	8.6	18	12.7%	-0.55 [-1.09, -0.02]	2008
Thies 2012 [41]	0.36	9.7	68	-0.54	11.9	76	22.2%	0.08 [-0.25, 0.41]	2012
Zeng 2013 [42]	-26.84	38.82	58	-12.07	38.29	59	19.9%	-0.38 [-0.75, -0.01]	2013
Deplanque 2016 [45]	-1	7	75	0	7	71	22.3%	-0.14 [-0.47, 0.18]	2016
**Total (95% CI)**			**311**			**270**	**100.0%**	**-0.15 [-0.38, 0.08]**	

Heterogeneity: Tau² = 0.03; Chi² = 8.77, df = 5 (P = 0.12); I² = 43%
Test for overall effect: Z = 1.30 (P = 0.19)

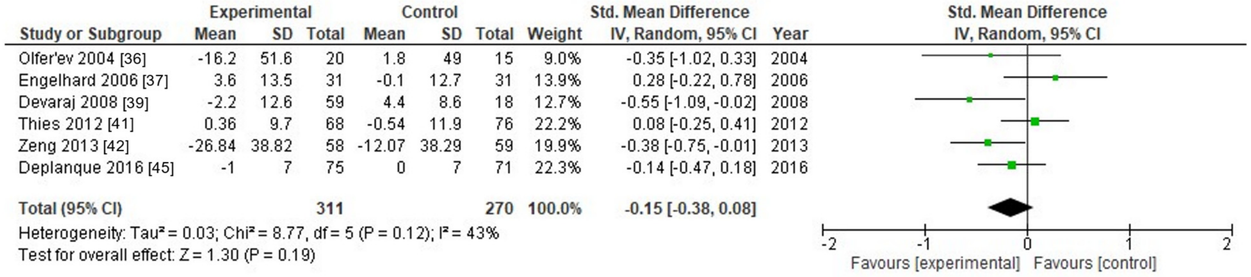

(b) Not supplement -type test foods

Study or Subgroup	Experimental Mean	Experimental SD	Experimental Total	Control Mean	Control SD	Control Total	Weight	Std. Mean Difference IV, Random, 95% CI	Year
Tsitsimpikou 2014 [44]	-6	40.8	15	-4	50	12	16.0%	-0.04 [-0.80, 0.72]	2014
Chernyshova 2019 [46]	4.32	11.44	10	1.08	10.87	10	11.9%	0.28 [-0.60, 1.16]	2019
Nishimura 2019 [47]	-0.7	4.3	49	0.4	4.5	49	58.5%	-0.25 [-0.65, 0.15]	2019
Takagi 2020 (High lutein arm) [49]	-4.7	10.09	7	-0.7	5.83	7	8.1%	-0.45 [-1.52, 0.61]	2020
Takagi 2020 (Low lutein arm) [49]	-1	10.54	5	6.6	9.3	5	5.5%	-0.69 [-1.99, 0.61]	2020
**Total (95% CI)**			**86**			**83**	**100.0%**	**-0.19 [-0.50, 0.11]**	

Heterogeneity: Tau² = 0.00; Chi² = 2.11, df = 4 (P = 0.71); I² = 0%
Test for overall effect: Z = 1.25 (P = 0.21)

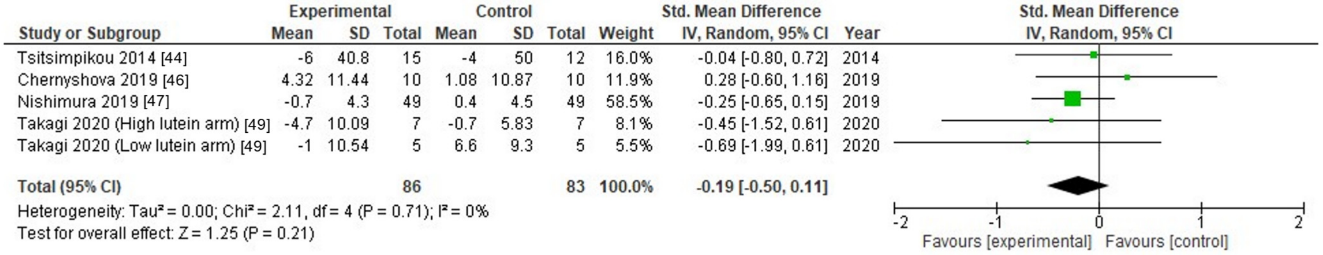

(c) Intake period ≤ 8 weeks

Study or Subgroup	Experimental Mean	Experimental SD	Experimental Total	Control Mean	Control SD	Control Total	Weight	Std. Mean Difference IV, Random, 95% CI	Year
Engelhard 2006 [37]	3.6	13.5	31	-0.1	12.7	31	22.5%	0.28 [-0.22, 0.78]	2006
Devaraj 2008 [39]	-2.2	12.6	59	4.4	8.6	18	20.5%	-0.55 [-1.09, -0.02]	2008
Deplanque 2016 [45]	-1	7	75	0	7	71	35.9%	-0.14 [-0.47, 0.18]	2016
Chernyshova 2019 [46]	4.32	11.44	10	1.08	10.87	10	9.5%	0.28 [-0.60, 1.16]	2019
Takagi 2020 (High lutein arm) [49]	-4.7	10.09	7	-0.7	5.83	7	6.8%	-0.45 [-1.52, 0.61]	2020
Takagi 2020 (Low lutein arm) [49]	-1	10.54	5	6.6	9.3	5	4.8%	-0.69 [-1.99, 0.61]	2020
**Total (95% CI)**			**187**			**142**	**100.0%**	**-0.14 [-0.43, 0.16]**	

Heterogeneity: Tau² = 0.04; Chi² = 6.86, df = 5 (P = 0.23); I² = 27%
Test for overall effect: Z = 0.93 (P = 0.35)

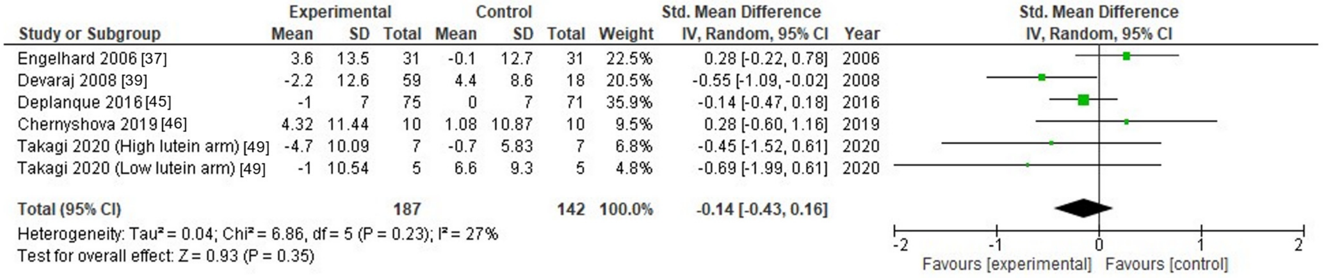

(d) Intake period > 8 weeks

Study or Subgroup	Experimental Mean	Experimental SD	Experimental Total	Control Mean	Control SD	Control Total	Weight	Std. Mean Difference IV, Random, 95% CI	Year
Olfer'ev 2004 [36]	-16.2	51.6	20	1.8	49	15	8.2%	-0.35 [-1.02, 0.33]	2004
Thies 2012 [41]	0.36	9.7	68	-0.54	11.9	76	34.2%	0.08 [-0.25, 0.41]	2012
Zeng 2013 [42]	-26.84	38.82	58	-12.07	38.29	59	27.6%	-0.38 [-0.75, -0.01]	2013
Tsitsimpikou 2014 [44]	-6	40.8	15	-4	50	12	6.5%	-0.04 [-0.80, 0.72]	2014
Nishimura 2019 [47]	-0.7	4.3	49	0.4	4.5	49	23.4%	-0.25 [-0.65, 0.15]	2019
**Total (95% CI)**			**210**			**211**	**100.0%**	**-0.17 [-0.36, 0.03]**	

Heterogeneity: Tau² = 0.00; Chi² = 4.07, df = 4 (P = 0.40); I² = 2%
Test for overall effect: Z = 1.68 (P = 0.09)

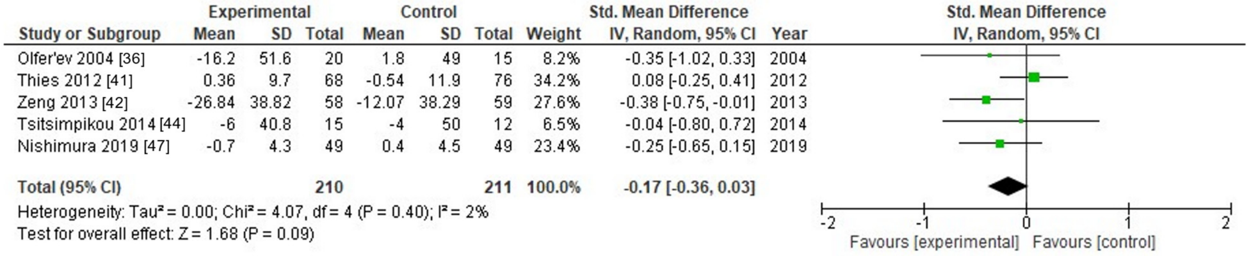

Supplementary Figure S1. (continued on next page)

(e) Healthy subjects

Study or Subgroup	Experimental Mean	Experimental SD	Experimental Total	Control Mean	Control SD	Control Total	Weight	Std. Mean Difference IV, Random, 95% CI	Year
Devaraj 2008 [39]	-2.2	12.6	59	4.4	8.6	18	17.0%	-0.55 [-1.09, -0.02]	2008
Deplanque 2016 [45]	-1	7	75	0	7	71	46.0%	-0.14 [-0.47, 0.18]	2016
Chernyshova 2019 [46]	4.32	11.44	10	1.08	10.87	10	6.3%	0.28 [-0.60, 1.16]	2019
Nishimura 2019 [47]	-0.7	4.3	49	0.4	4.5	49	30.8%	-0.25 [-0.65, 0.15]	2019
**Total (95% CI)**			**193**			**148**	**100.0%**	**-0.22 [-0.44, 0.00]**	

Heterogeneity: Tau² = 0.00; Chi² = 2.95, df = 3 (P = 0.40); I² = 0%
Test for overall effect: Z = 1.94 (P = 0.05)

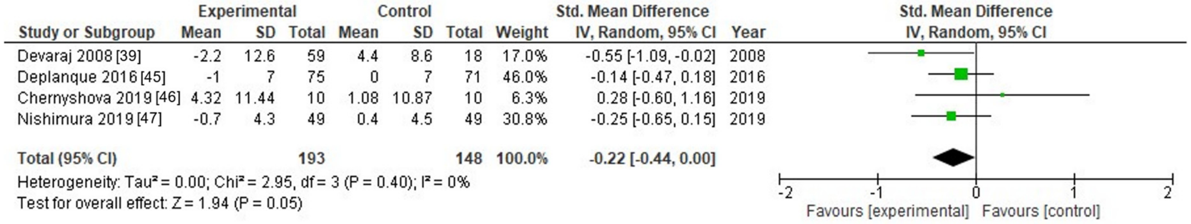

(f) Not healthy subjects

Study or Subgroup	Experimental Mean	Experimental SD	Experimental Total	Control Mean	Control SD	Control Total	Weight	Std. Mean Difference IV, Random, 95% CI	Year
Olfer'ev 2004 [36]	-16.2	51.6	20	1.8	49	15	10.3%	-0.35 [-1.02, 0.33]	2004
Engelhard 2006 [37]	3.6	13.5	31	-0.1	12.7	31	16.9%	0.28 [-0.22, 0.78]	2006
Thies 2012 [41]	0.36	9.7	68	-0.54	11.9	76	30.4%	0.08 [-0.25, 0.41]	2012
Zeng 2013 [42]	-26.84	38.82	58	-12.07	38.29	59	26.5%	-0.38 [-0.75, -0.01]	2013
Tsitsimpikou 2014 [44]	-6	40.8	15	-4	50	12	8.4%	-0.04 [-0.80, 0.72]	2014
Takagi 2020 (High lutein arm) [49]	-4.7	10.09	7	-0.7	5.83	7	4.5%	-0.45 [-1.52, 0.61]	2020
Takagi 2020 (Low lutein arm) [49]	-1	10.54	5	6.6	9.3	5	3.1%	-0.69 [-1.99, 0.61]	2020
**Total (95% CI)**			**204**			**205**	**100.0%**	**-0.11 [-0.34, 0.12]**	

Heterogeneity: Tau² = 0.02; Chi² = 7.41, df = 6 (P = 0.28); I² = 19%
Test for overall effect: Z = 0.92 (P = 0.36)

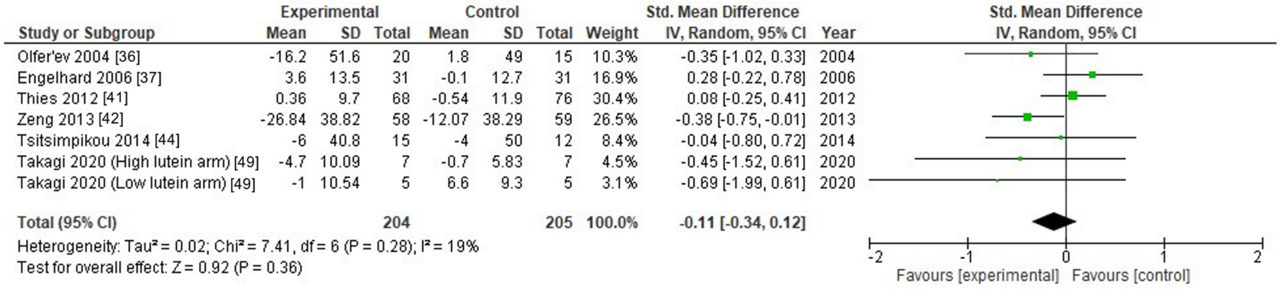

Supplementary Figure S1. Subgroup meta-analyses of the effects of lycopene on fasting blood glucose: (a) supplement-type test foods (*n* = 6 studies), (b) not supplement-type test foods (*n* = 5 trial arms), (c) intake period ≤ 8 weeks (*n* = 6 trial arms), (d) intake period > 8 weeks (*n* = 5 studies), (e) healthy subjects (*n* = 4 studies) and (f) not healthy subjects (*n* = 7 trial arms). The green squares represent the standardized mean difference in each study. The black diamonds represent the pooled effects in each meta-analysis. Std., standardized; SD, standard deviation; IV, inverse variance; CI, confidence interval.
